# Intra- and inter-observer reliability and repeatability of the metatarsus adductus angle in childhood: A concordance study

**DOI:** 10.1007/s00247-025-06375-3

**Published:** 2025-08-25

**Authors:** Eduardo Pérez-Boal, Carlos Martin-Villa, Ricardo Becerro-de-Bengoa-Vallejo, Marta Elena Losa-Iglesias, Bibiana Trevissón-Redondo, Daniel López-López, César Calvo-Lobo, David Rodríguez-Sanz, Eva María Martínez-Jiménez, Israel Casado-Hernández

**Affiliations:** 1 Departamento de Enfermería y Fisioterapia, Facultad de Ciencias de Salud. Campus de Ponferrada, Universidad de León, León, Spain; 2 Grupo de investigación FEBIO, Universidad Complutense de Madrid, Madrid, Spain; 3 Departamento de Enfermería, Facultad de Enfermería, Fisioterapia y Podología, Plaza de Ramón y Cajal, s/n, Edificio de la Facultad de Medicina, Pabellón 1º, 3ª Planta, Ciudad Universitaria, Madrid, 28040 Spain; 4 Facultad de Ciencias de la Salud, Campus de Alcorcón, Universidad Rey Juan Carlos, Madrid, Spain; 5 Research, Health and Podiatry Group, Department of Health Sciences, Faculty of Nursing and Podiatry, Industrial Campus of Ferrol, Universidade da Coruña, Ferrol, A Coruña, Spain

**Keywords:** Child, Metatarsus varus, Radiology, Reproducibility of results

## Abstract

**Background:**

Metatarsus adductus is a relatively common congenital foot deformity often unrecognized at birth. There is adduction of the metatarsals, supination of the subtalar joint, and plantar flexion of the first ray.

**Objective:**

The objective of this study was to utilize the most commonly used methods for assessing metatarsus adductus deformity in children and to identify the measurements with the least intra- and inter-observer variability.

**Materials and methods:**

All consecutive patients between 2 and 9 years of age with metatarsus adductus by roentgenographic evaluation on weight-bearing dorsoplantar images were included in this cross-sectional study. Measurements included 32 radiographs, 18 feet from 14 girls with a mean age of 3.73 ± 2.53 years, and 14 feet corresponding to 11 boys with a mean age of 5.21 ± 2.15 years. We identified and assessed all radiographic measurements to evaluate the metatarsus adductus with the different measurements commonly used today, including the Berg, Kilmartin, Laaveg & Ponseti, rearfoot, Root, and Simons’ angles.

**Results:**

The reliability of the variables measured in 32 weight-bearing dorsoplantar foot radiographs showed excellent reliability ranging in Laaveg-Ponseti and rearfoot angles with low standard error of measurement (SEM), coefficient of variation (CV), and minimum detectable change (MDC) with intraclass correlation coefficient (ICC) higher than 0.990. Kilmartin, Root, and Simons’ angles showed good intra- and inter-observer reliability, with ICC values greater than 0.900; however, they also exhibited systematic differences between sessions or between observers (*P* < 0.05), or a high MDC value, which may limit their reproducibility.

**Conclusion:**

Based on the results obtained in this study from the different measurements performed, the rearfoot measurement and the method proposed by Laaveg and Ponseti can be considered the most reliable, as they showed the highest intra- and inter-observer reliability.

**Graphical Abstract:**

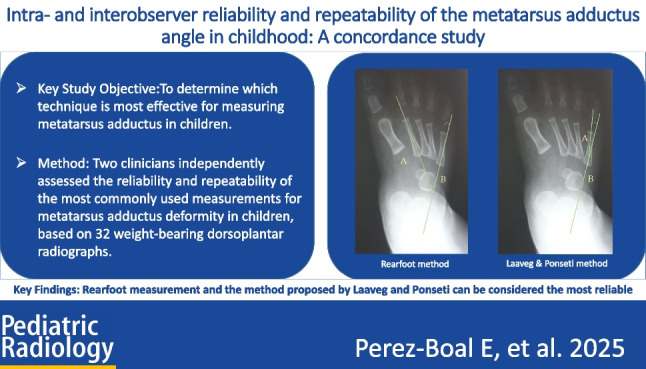

## Introduction

Metatarsus adductus is a congenital foot deformity in the transverse plane in which the metatarsals deviate medially to the midfoot [1, 2]. There is adduction of the metatarsals, plantar flexion of the first ray, and supination of the subtalar joint [3]. Metatarsus adductus is characterized by a convex lateral border of the foot, a prominent styloid process, and an inward slant of the forefoot. Unlike other congenital foot deformities, it exclusively affects the forefoot [4]. Metatarsus adductus may be associated with clubfeet in the pediatric population or even adolescents and adults [5–7].

The epidemiology of metatarsus adductus varies from 0.1% to 12% [8–10]. Its incidence among Caucasians is 1 per 1,000 live births [11]. Racial differences occur, with an incidence in the Māori population of 7 per 1,000 live births [12].

Previous studies have indicated that females had a higher incidence of the deformity, with a ratio of 3:1 [13]. Recently, studies have reported a higher incidence of males compared to females (2.0–2.5) [14]. In about 50% of cases, the deformity is bilateral, and the right foot is affected more subtly than the left foot [15].

Most children with metatarsus adductus experience complete spontaneous resolution [16]. However, other studies indicate that up to 14% of affected children may have the deformity persist into adulthood [17, 18]. Relevant evidence suggests that when spontaneous resolution does not occur, further treatment may be necessary. Initially, this will be conservative, involving stretching exercises, foot orthotics, and serial splinting, while more severe cases or those with greater rigidity may require surgical intervention [4, 16, 19–24].

While numerous assessment techniques have been published to quantify the presence of metatarsus adductus, there is scant research evaluating the reliability of measurement techniques. This is potentially problematic for clinicians, as quantification of the condition drives treatment and management decisions [25].

Reliability is a fundamental problem for measurement in all sciences [26–28]. The present study aims to assess intra- and inter-observer repeatability and reliability using the measuring system most used in the literature [25] adapted to children’s feet to find the most suitable valuation of metatarsus adductus. Due to the limited research comparing intra- and inter-observer measurements, we aim to present the most effective measures for a complete assessment of metatarsus adductus. We consider this important in helping physicians become highly efficient when carrying out radiological evaluations.

No previous publications evaluate all the methods currently used to measure the metatarsus adductus deformity in children. The primary objective of this study was to utilize the most commonly used methods for assessing metatarsus adductus deformity in children and to identify the measurements with the least intra- and inter-observer variability.

## Materials and methods

### Study design

A reliability study was performed to determine the intra-rater and inter-rater reliability of six different measurements of metatarsus adductus in two sessions.

### Sample size calculation

Sample size calculation was performed by *t*-test family using correlation statistical tests of the G*Power 3.1.9.7 software version, with normal distribution. A two-tailed hypothesis, a large effect size of 0.50, an *α* error probability of 0.05, with a *β* level of 20% and a desired power analysis of 80% (1-*β* error probability) were used for the sample size calculations. As a result, a total sample size of 26 participants was deemed appropriate. A total of 32 participants were recruited by a consecutive sampling method using a successive and non-randomized simple method. Subjects were enlisted from the foot and ankle clinical unit at the CEMTRO Hospital (Madrid, Spain). The Research Ethical Committee at Madrid University of Rey Juan Carlos (URJC) approved this study with internal register number 0212201600117. Ethical standards for human experimentation of the Declaration of Helsinki and local regulations were observed [29]. In addition, written informed consent was obtained for all registered participants. Since all participants were minors, informant consent was obtained from their parents. The sample consisted of patients from the foot and ankle clinical unit at the CEMTRO Hospital of Madrid from September 2021 to January 2022. All consecutive patients over 2 years and under 9 years of age with metatarsus adductus by roentgenographic evaluation were included in this cross-sectional study. Measurements included 32 radiographs, 18 feet from 14 girls with a mean age of 3.73 ± 2.53 years, and 14 feet corresponding to 11 boys with a mean age of 5.21 ± 2.15 years.

A Discovery XR656 Plus X-ray system (General Electric Research, Milwaukee, WI) was used to take foot images at a source-to-image distance of 100 cm and was set to 60 kVp and 2.5 mAs. Using PACS (IMPAX; Agfa Healthcare, Mortsel, BE), we transferred radiographic images using professional digital image management. Radiographic measurements were performed using a digital radiographic imaging and measurement system AutoCad 2019 (Autodesk Inc., San Rafael, CA). Dorsoplantar radiographs for weight-bearing conditions were performed independently for each foot, with the patients standing with the knee extended. The foot was aligned to prevent internal or external rotation of the leg and was pointed straight forward, parallel to the sagittal plane [30, 31]. The X-ray beam was inclined 15° in the dorsal-plantar direction centered on the second tarsometatarsal joint [32–38].

We reviewed 46 weight-bearing dorsoplantar foot radiographs and excluded 14, which did not meet the inclusion criteria. Inclusion criteria included patients over 2 years and under 8 years of age [39], with no history of a previous forefoot, midfoot, or rearfoot surgery, and no evidence of foot trauma that could affect foot anatomy. Exclusion criteria included neurologic disease, lower limb malformation, fracture, or prior surgery of the foot or lower limb, neuromuscular disorders, and foot scar [40]. We established the metatarsus adductus criteria > 35° [1].

### Metatarsus adductus measurements

**Fig. 1:**
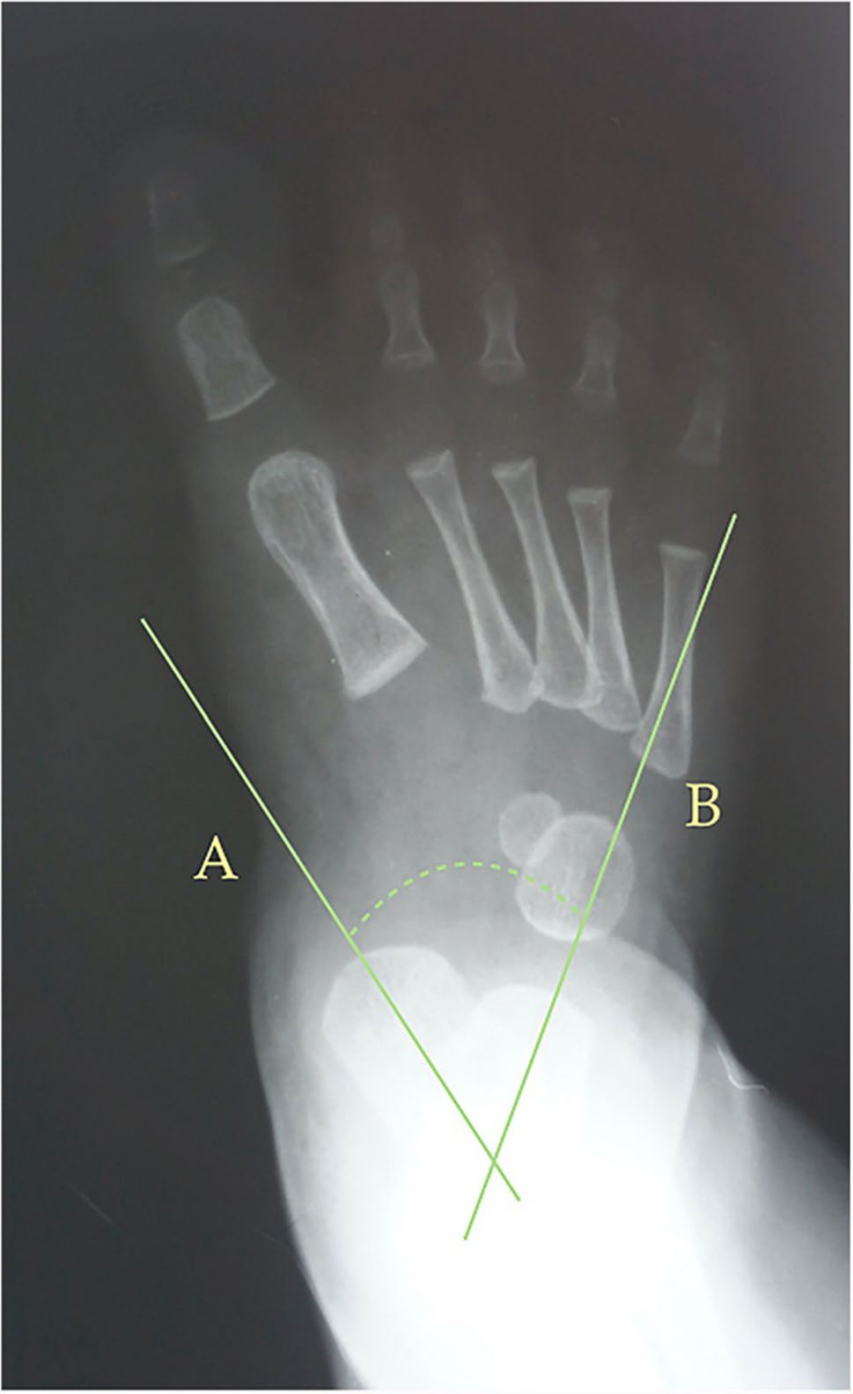
Dorsoplantar weighting-bearing Berg’s angle is the angle between the longitudinal axis of the talus, line (**A**), and the longitudinal axis of the calcaneus bone, line (**B**)

**Fig. 2:**
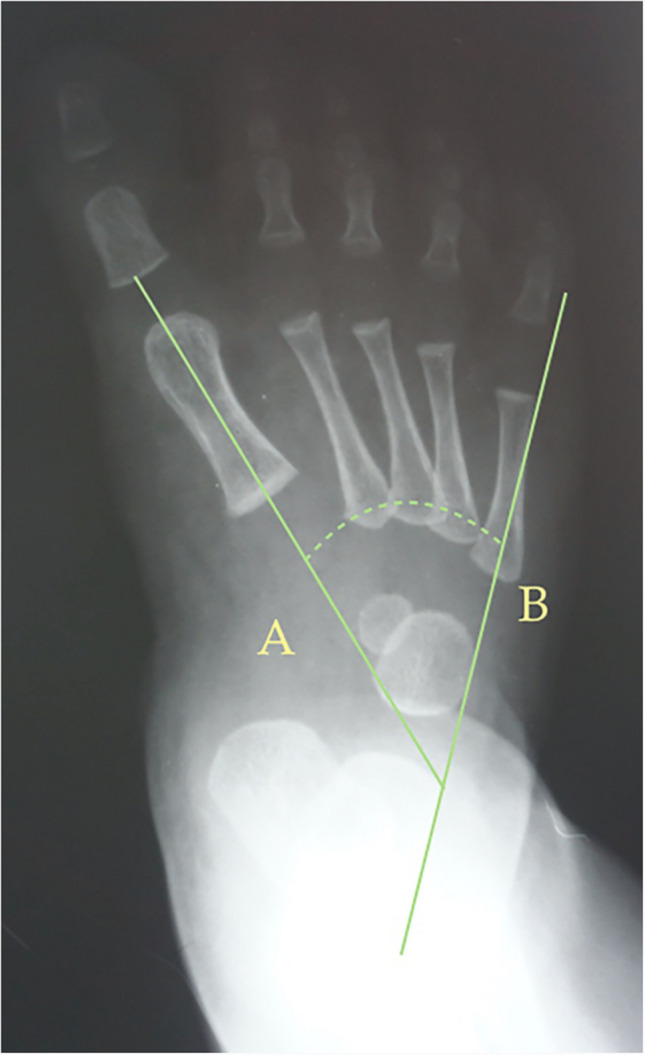
Dorsoplantar weight-bearing Kilmartin’s angle is the angle between the longitudinal axis of the first metatarsus, line (**A**), and a parallel line to the lateral border of the calcaneum, line (**B**)

**Fig. 3:**
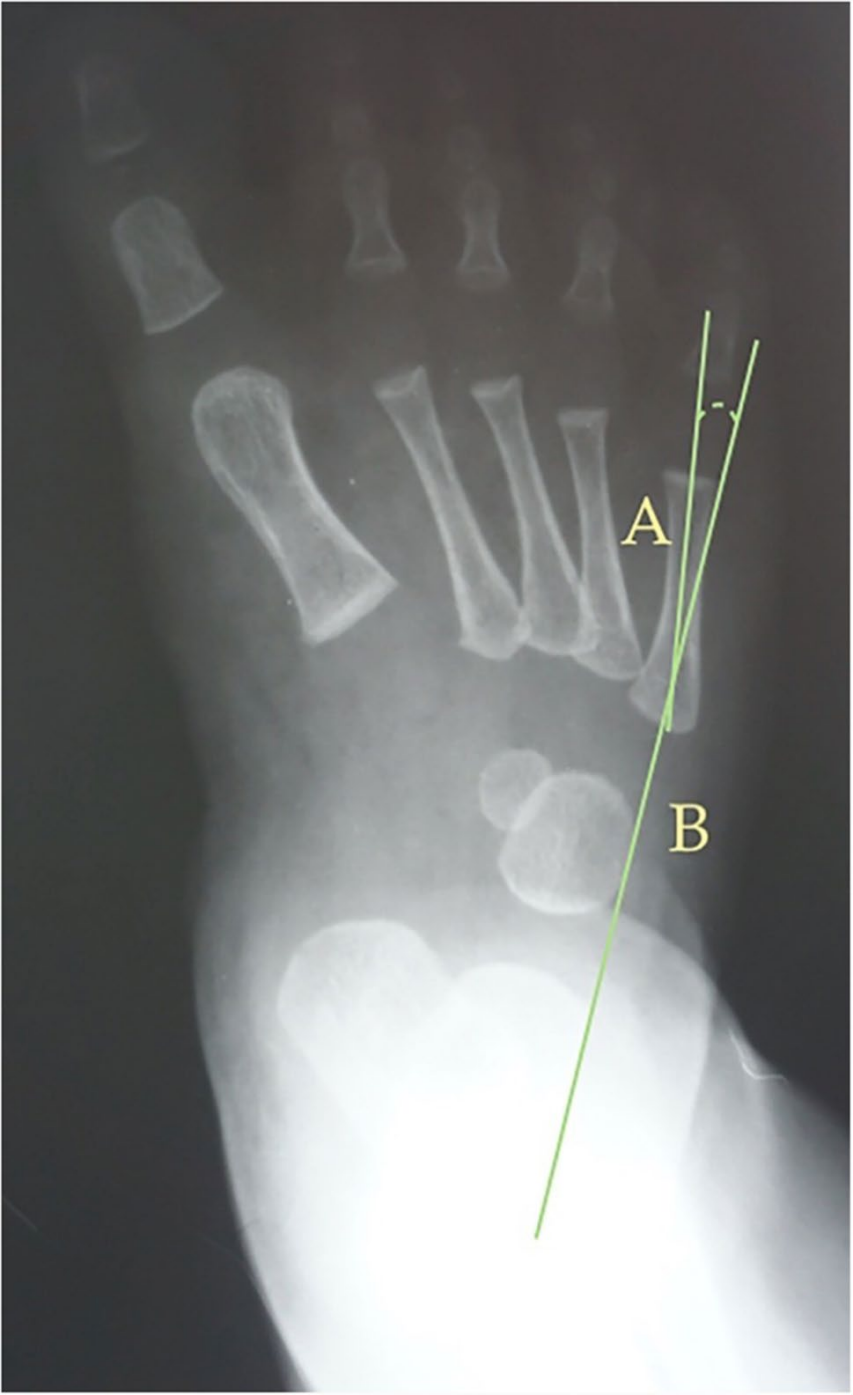
Dorsoplantar weight-bearing Laaveg & Ponseti’s angle is the angle between a line parallel to the lateral surface of calcaneum bone, line (**B**), and the longitudinal axis of the fifth metatarsal bone, line (**A**)

**Fig. 4:**
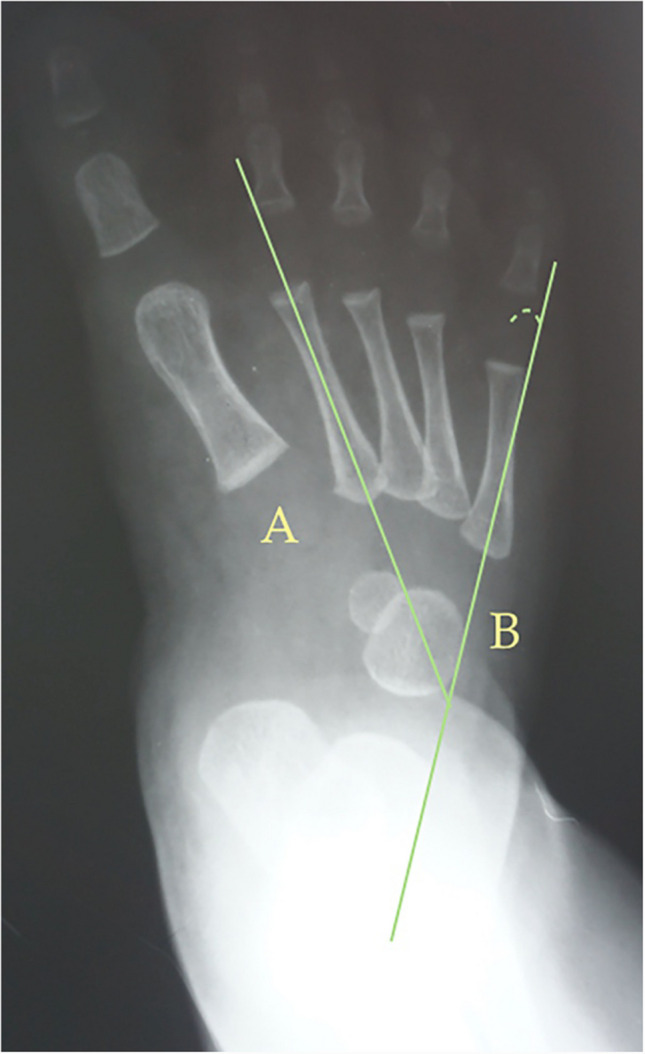
Dorsoplantar weight-bearing rearfoot angle is the angle between a parallel line to the lateral border of the calcaneum, line (**B**), and the longitudinal axis of the second metatarsal bone, line (**A**)

**Fig. 5:**
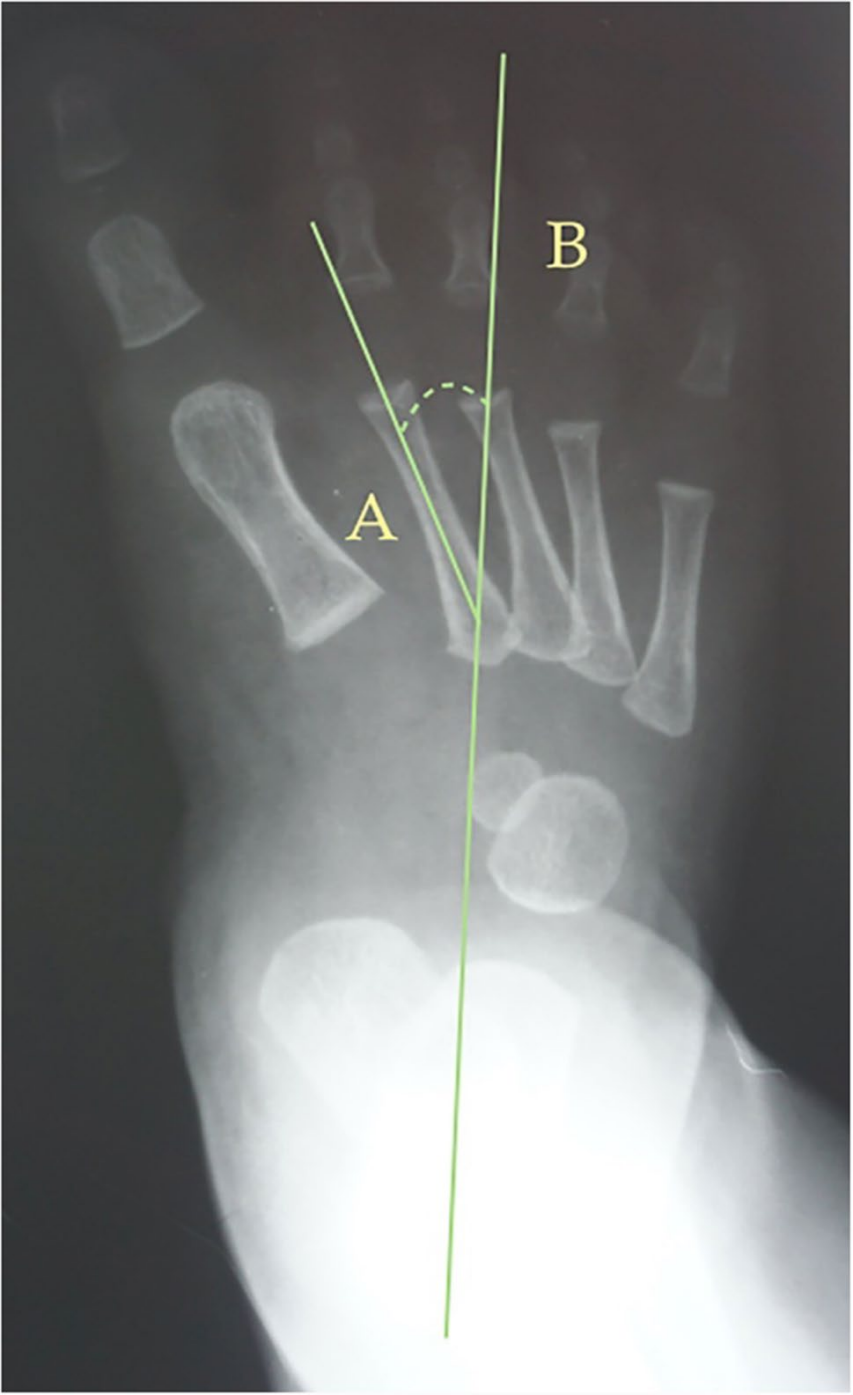
Dorsoplantar weight-bearing Root’s angle measures the adduction of the forefoot by using an angle formed between the longitudinal axis of the second metatarsus, line (**A**), and the longitudinal axis of the rearfoot, line (**B**)

**Fig. 6:**
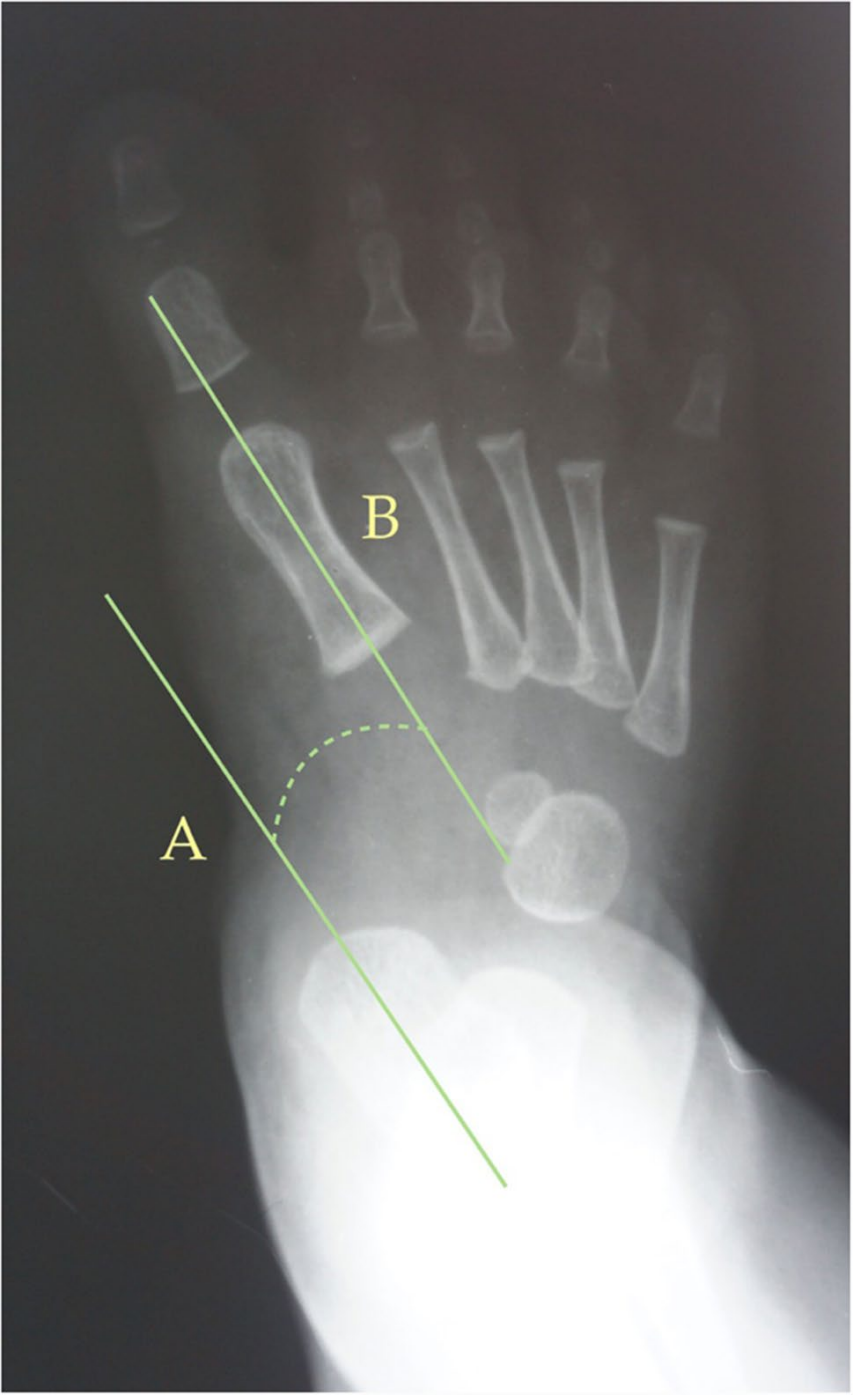
Dorsoplantar weight-bearing Simons’ angle is the angle between the longitudinal axis of the first metatarsal, line (**B**), and a perpendicular line, line (**A**), of the articular surface of the talar head – longitudinal axis of the talus

To assess metatarsus adductus, different methods have been reported including Berg’s method [40] as shown in Fig. 1, Kilmartin’s angle [41] as shown in Fig. 2, Laaveg & Ponseti’s angle as shown in Fig. 3 [42], rearfoot angle as shown in Fig. 4 [43–46], Root’s angle as shown in Fig. 5 [3], and Simons’ angle as shown in Fig. 6 [47].

The following angles were examined on each radiograph: Berg angle [40] (Fig. 1) is the angle between the longitudinal axis of the talus, line (A), and the longitudinal axis of the calcaneus bone, line (B). Kilmartin’s angle [41] (Fig. 2) is the angle between the longitudinal axis of the first metatarsus, line (A), and a parallel line to the lateral border of the calcaneum, line (B). Laaveg & Ponseti’s angle [42] (Fig. 3) is the angle between a line parallel to the lateral surface of calcaneum bone, line (B), and the longitudinal axis of the fifth metatarsal bone, line (A). Rearfoot angle [43–46] (Fig. 4) is the angle between a parallel line to the lateral border of the calcaneum, line (B), and the longitudinal axis of the second metatarsal bone, line (A). Root’s angle (Fig. 5) [3] measures the adduction of the forefoot by using an angle formed between the longitudinal axis of the second metatarsus, line (A), and the longitudinal axis of the rearfoot, line (B). Simons’ angle [47] (Fig. 6) is the angle between the longitudinal axis of the first metatarsal, line (B), and a perpendicular line, line (A), of the articular surface of the talar head – longitudinal axis of the talus.

### Reliability study protocol

Two physicians E.P-B and C.M-V with more than 20 years of radiology experience independently conducted all measurements for each radiograph six times, three times during the first session, and three additional times in a second session. The period between both sessions was 1 week. Before each measurement, the order of the radiographs was randomized. Both clinicians were trained on an initial set of three radiographs under the direction of another senior clinician with more than 30 years of radiology experience.

### Statistical analysis

Quantitative values were described as mean and standard deviation (SD), lower and upper limits for a 95% confidence interval (CI), and median and interquartile range.

All variables were examined using the Kolmogorov–Smirnov test, and data were normally distributed if *P* > 0.05. Parametric independent Student *t*-tests were performed to determine whether differences were statistically significant, and non-parametric *U* Mann–Whitney tests were applied when non-normal distribution was shown.

Reliability between measurements was determined using the intraclass correlation coefficient (ICC), absolute agreement, two-way random effects, multiple raters/measurements (ICC 2,k), and a mean of two trials for each test session on each radiograph was used to calculate intersession reliability. ICC values were interpreted as poor (ICC < 0.40), fair (ICC = 0.40–0.59), good (ICC = 0.60–0.74), and excellent (ICC = 0.75–1.0) [48].

Portney and Watkins’s recommendations state that clinical measures with an ICC greater than 0.90 improve the probability that the measure is valid [49].

Coefficient of variation (CV) was analyzed for the absolute comparison of parameters and was calculated to test the intra-session reliability as the mean normalized to the SD. A higher CV value shows a higher dispersion.

In addition, Pearson’s correlation coefficient (*r*) was also used and categorized as weak (*r* = 0.00–0.40), moderate (*r* = 0.41–0.69), and strong (*r* = 0.70–1.00) [48].

The 95% limits of agreement (LoA) between sessions and devices expressed the degree of error proportional to the mean of the measurement units. These statistics were calculated using the methods described by Bland and Altman [50]. If the differences between the measurements tended to agree, the results were close to zero.

Standard errors of measurement were calculated to measure the range of error of each parameter. The standard error of measurement (SEM) was calculated from the ICCs and SDs for each of the three measurements according to the formula SEM = SD × sqrt (1 − ICC).

The minimum detectable change at a confidence level of 95%, which reflects the magnitude of change necessary to provide confidence that a change is not the result of random variation or measurement error, was calculated from the SEM values by the following formula MDC = $\sqrt{2}\times 1.96\times \text{SEM}$. Both MDC and SEM were analyzed according to Bland and Altman [50].

Statistical significance was established by a *P* < 0.05, with CI of 95%, and analyses were performed using SPSS 25.0 software (SPSS Inc., Chicago, IL).

## Results

A total of 32 participants were recruited. Descriptive data of the age variable had a normal distribution (*P* = 0.100) as shown in Table 1.

**Table 1:** Patient demographics

Variables	Total (*N* = 32)		Male (*n* = 14)			Female (*n* = 18)			*P*-value
Descriptive data	Mean ± SD (95% CI)	Median (IR)	Mean ± SD (95% CI)	Median (IR)	***P*** K-S	Mean ± SD (95% CI)	Median (IR)	***P*** K-S	
Age	4.40 ± 2.44 (3.52–5.28)	4.50 (5.00)	5.21 ± 2.15 (3.97–6.45)	5.00 (4.00)	0.053	3.77 ± 2.53 (2.51–5.03)	2.00 (4.25)	0.001	0.100

*CI*, confidence interval; *IR*, interquartile range; *N*, sample size; *p K-S*, Kolmogorov–Smirnov test and *P* > 0.05 considered normal distribution; *P*-value, from *U* Mann–Whitney for independent group. Statistical significance for a *P*-value < 0.05, with a 95% CI; *SD*, standard deviation

Table 2 shows the first observer’s analysis of the reliability of the metatarsus adductus angle. Reliability for the metatarsus adductus angle on the first and second sessions was excellent with ICC > 0.920 for all methods except Simons’ angle. Reliability intersession values ranged from [ICC = 0.909 (0.814–0.956)] to [ICC = 0.982 (0.963–0.991)]; SEM and CV values had very low measurements as did MDC, except for Kilmartin’s angle = 5.049 and Simons’ angle = 12.692. There were systematic differences between sessions on Root’s angle and Simons’ angle (< 0.05).

**Table 2:** Analysis of metatarsus adductus angle measurements between the first and second session by observer 1

	**Observer 1 metatarsus adductus measurements**																		
Sessions	**First session**						**Second session**						**Intersession**						
Classification	Mean (SD) (CI 95%)	Median (CI 95%)	ICC (CI 95%)	SEM	CV	MDC	Mean (SD) (CI 95%)	Median (CI 95%)	ICC (CI 95%)	SEM	CV	MDC	Mean (SD) (CI 95%)	Median (CI 95%)	ICC (CI 95%)	SEM	CV	MDC	*P*-value
Berg	39.22 ± 4.44 (37.62–40.82)	37.34 (36.53–36.68)	0.949 (0.990–0.997)	0.318	0.113	0.881	39.23 ± 4.14 (37.74–40.72)	37.50 (36.84–38.35)	0.926 (0.932–0.980)	1.125	0.105	3.118	39.22 ± 4.15 (37.73–40.72)	37.48 (36.81–38.88)	0.931 (0.859–0.966)	1.086	0.106	3.01	0.471
Kilmartin	29.57 ± 12.74 (24.97–34.16)	25.66 (21.41–32.07)	0.998 (0.996–0.991)	0.525	0.431	1.456	29.65 ± 10.90 (25.71–33.58)	28.73 (24.10–31.91)	0.972 (0.950–0.985)	1.822	0.367	5.049	29.61 ± 11.34 (25.52–33.70)	27.47 (23.09–32.04)	0.909 (0.814–0.956)	3.407	0.383	9.44	0.134
Laaveg & Ponseti	8.26 ± 7.31 (5.63–10.90)	5.34 (3.41–10.27)	0.998 (0.966–0.999)	0.319	0.885	0.884	7.70 ± 7.08 (5.14–10.25)	5.04 (2.58–6.89)	0.997 (0.995–0.998)	0.375	0.920	1.039	7.98 ± 7.08 (5.42–10.53)	5.28 (3.39–8.54)	0.965 (0.930–0.983)	1.308	0.887	3.63	0.257
Rearfoot	22.41 ± 11.65 (18.20–26.61)	20.62 (15.69–23.25)	0.998 (0.997–0.999)	0.404	0.520	1.119	23.11 ± 10.13 (19.46–26.76)	20.44 (17.46–24.17)	0.997 (0.995–0.998)	0.486	0.438	1.347	22.76 ± 10.82 (18.85–26.66)	20.61 (16.14–22.56)	0.982 (0.963–0.991)	1.453	0.476	4.03	0.129
Root	13.49 ± 10.64 (9.65–17.32)	10.13 (7.53–12.71)	0.998 (0..997–0.999)	0.412	0.789	1.142	15.90 ± 11.19 (11.86–19.94)	11.09 (9.68–15.93)	0.977 (0.956–0.988)	1.679	0.703	4.655	14.69 ± 10.68 (10.84–18.55)	10.44 (9.10–13.84)	0.945 (0.857–0.976)	2.505	0.727	6.94	0.003
Simons	14.44 ± 12.45 (9.95–18.93)	10.03 (7.70–13.36)	0.999 (0.998–0.999)	0.305	0.863	0.846	16.59 ± 12.46 (12.09–21.08)	11.93 (10.81–17.02)	0.865 (0.758–0.929)	4.579	0.751	12.692	15.51 ± 12.11 (11.15–19.88)	11.48 (9.53–14.59)	0.935 (0.862–0.969)	3.071	0.781	8.51	0.008

*CI*, confidence interval; *CV*, coefficient of variation; *ICC*, intraclass correlation coefficient; *IR*, interquartile range; *MDC*, minimum detectable change; *P*-value from Wilcoxon signed-rank test; statistical significance for a *P*-value <0.05, with a 95% CI; *SD*, standard deviation; *SEM*, standard error of measurement

Table 3 shows the analysis of the reliability of the metatarsus adductus angle by the second observer. Excellent values were obtained in the first and second sessions with ICC > 0.990 for most methods except Root’s angle [ICC = 0.890 (0.482–0.962)]. Reliability intersession values ranged from [ICC = 0.994 (0.986–0.997)] to [ICC = 0.999 (0.998–0.999)]; SEM was low for all methods except Simons’ angle with SEM = 4.579; MDC on Simons’ angle was 9.765, while the rest of the methods were low. CV was low for all methods. We found systematic differences between sessions on Berg’s angle and Kilmartin’s angle (*P* < 0.05).

**Table 3:** Analysis of metatarsus adductus angle measurements between the first and second session by observer 2

Observer 2 metatarsus adductus measurements																			
Sessions	**First session**						**Second session**						**Intersession**						
Classification	Mean (SD) (CI 95%)	Median (CI 95%)	ICC (CI 95%)	SEM	CV	MDC	Mean (SD) (CI 95%)	Median (CI 95%)	ICC (CI 95%)	SEM	CV	MDC	Mean (SD) (CI 95%)	Median (CI 95%)	ICC (CI 95%)	SEM	CV	MDC	*P*-value
Berg	38.95 ± 4.32 (37.39–40.51)	37.01 (36.22–38.85)	0.990 (0.983–0.995)	0.419	0.111	1.161	39.22 ± 4.44 (37.62–40.82)	37.34 (36.53–38.68)	0.994 (0.990–0.997)	1.125	0.318	0.881	39.08 ± 4.37 (37.51–40.66)	37.21 (36.45–38.76)	0.994 (0.986–0.997)	0.336	0.112	0.931	0.006
Kilmartin	29.33 ± 12.63 (24.77–33.89)	25.97 (20.75–31.75)	0.999 (0.998–0.999)	0.379	0.431	1.051	29.57 ± 12.74 (24.97–34.16)	25.66 (21.41–32.07)	0.998 (0.996–0.999)	1.822	0.526	1.457	29.45 ± 12.68 (24.87–34.02)	25.81 (21.05–31.96)	0.999 (0.998–0.999)	0.284	0.431	0.786	0.011
Laaveg & Ponseti	8.30 ± 7.32 (5.66–10.94)	5.04 (3.73–10.74)	0.998 (0.996–0.999)	0.302	0.882	0.837	8.26 ± 7.31 (5.63–10.90)	5.34 (3.41–10.27)	0.998 (0.996–0.999)	0.375	0.319	0.884	8.28 ± 7.31 (5.64–10.92)	5.17 (3.56–10.60)	0.999 (0.998–0.999)	0.179	0.883	0.497	0..587
Rearfoot	22.44 ± 11.95 (18.12–26.75)	20.72 (15.81–22.65)	0.999 (0.998–0.999)	0.378	0.533	1.048	22.41 ± 11.65 (18.20–26.61)	20.62 (15.69–23.25)	0.998 (0.997–0.999)	0.486	3.688	1.222	22.42 ± 11.80 (18.17–26.68)	20.68 (15.72–22.95)	0.999 (0.998–0.999)	0.334	0.526	0.925	0.653
Root	13.55 ± 10.80 (9.65–17.44)	9.84 (7.75–13.11)	0.998 (0.997–0.999)	0.374	0.797	1.037	13.49 ± 10.64 (9.65–17.32)	10.13 (7.53–12.71)	0.890 (0.482–0.962)	1.679	3.526	9.765	13.52 ± 10.71 (9.65–17.38)	9.99 (7.53–12.79)	0.999 (0.998–0.999)	0.240	0.793	0.664	0.783
Simons	14.28 ± 12.52 (9.77–18.80)	9.75 (7.25–12.59)	0.999 (0.998–0.999)	0.307	0.876	0.850	14.44 ± 12.45 (9.95–18.93)	10.03 (7.70–13.36)	0.999 (0.998–0.999)	4.579	0.305	0.846	14.36 ± 12.48 (9.86–18.86)	9.89 (7.55–13.15)	0.999 (0.998–0.999)	0.250	0.869	0.692	0.056

*CI*, confidence interval; *CV*, coefficient of variation; *ICC*, intraclass correlation coefficient; *IR*, interquartile range; *MDC*, minimum detectable change; *P*-value from Wilcoxon signed-rank test; statistical significance for a *P*-value <0.05, with a 95% CI; *SD*, standard deviation; *SEM*, standard error of measurement

Table 4 shows the analysis of reliability of metatarsus adductus angle measurements between observers. Excellent ICC values obtained between observers were ICC > 0.980 for all methods. Strong correlations found in all angles between the first and second observer ranged from *r* = 0.967 and *r* = 0.994, respectively. Low SEM and CV were found in all methods. MDC was low for all methods except Kilmartin’s angle with MDC = 4.668 and Simons’ angle with MDC = 4.281. The LoA were very low for all methods. There were no systematic inter-observer differences (*P* < 0.05).

**Table 4:** Analysis of reliability of metatarsus adductus angle measurements between observers 1 and 2

Observer	Observer 1 Intersession		Observer 2 Intersession		Inter-observer							
Classification	Mean (SD) (CI 95%)	Median (CI 95%)	Mean (SD) (CI 95%)	Median (CI 95%)	Mean (SD) (CI 95%)	ICC _(1–1)_ (CI 95%)	SEM	CV	MDC	LoA (CI 95%)	***P***-value *	r** (***P***-value)
Berg	39.22 ± 4.15 (37.73–40.72)	37.48 (36.81–38.88)	39.08 ± 4.37 (37.51–40.66)	37.21 (36.45–38.76)	39.15 ± 4.22 (37.63–40.68)	0.983 (0.965–0.991)	0.550	0.108	1.525	−0.14 (−2.31–2.03)	0..390	0.967 (< 0.001)
Kilmartin	29.61 ± 11.34 (25.52–33.70)	27.47 (23.09–32.04)	29.45 ± 12.68 (24.87–34.02)	25.81 (21.05–31.96)	29.53 ± 11.91 (25.23–33.83)	0.980 (0.960–0.990)	1.684	0.403	4.668	−0.15 (−6.74–6.42)	0.690	0.967 (< 0.001)
Laaveg & Ponseti	7.98 ± 7.08 (5.42–10.53)	5.28 (3.39–8.54)	8.28 ± 7.31 (5.64–10.92)	5.17 (3.56–10.60)	8.13 ± 7.17 (5.55–10.72)	0.991 (0.982–0.995)	0.669	0.881	1.853	0.30 (−2.29–2.90)	0.914	0.983 (< 0.001)
Rearfoot	22.76 ± 10.82 (18.85–26.66)	20.61 (16.14–22.56)	22.42 ± 11.80 (18.17–26.68)	20.68 (15.72–22.95)	22.59 ± 11.29 (18.52–26.66)	0.995 (0.990–0.997)	0.782	0.500	2.168	−0.33 (−3.38–2.70)	0.717	0.994 (< 0.001)
Root	14.69 ± 10.68 (10.84–18.55)	10.44 (9.10–13.84)	13.52 ± 10.71 (9.65–17.38)	9.99 (7.53–12.79)	14.11 ± 10.64 (10.27–17.94)	0.986 (0.964–0.994)	1.259	0.754	3.489	−1.17 (−5.50–3.15)	0.485	0.978 (< 0.001)
Simons	15.51 ± 12.11 (11.15–19.88)	11.48 (9.53–14.59)	14.36 ± 12.48 (9.86–18.86)	9.89 (7.55–13.15)	14.94 ± 12.21 (10.53–19.34)	0.984 (0.965–0.992)	1.544	0.817	4.281	−1.15 (−6.80–4.50)	0.368	0.972 (< 0.001)

*CI*, confidence interval; *ICC*, intraclass correlation coefficient; *LoA*, 95% limits of agreement; *MDC*, minimum detectable change; *SD*, standard deviation; *SEM*, standard error of measurement; *r*, **Spearman correlation coefficient; *P-value from *U* Mann–Whitney; statistical significance for a *P*-value < 0.05, with a 95% confidence interval

## Discussion

Various radiographic measurements have been developed to valuate metatarsus adductus [3, 40–46, 50, 51]; however, no research has compared the reliability and repeatability of standard methods for assessing radiographic metatarsus adductus in children. This study aims to identify the best reliability and repeatability methods of assessing radiographic metatarsus adductus and update the current guidelines regarding the intra- and inter-observer reliability of the standard measurement most currently used in radiographic metatarsus adductus assessment in childhood.

Intra- and inter-observer reliability of common angular measurements of various foot disorders has been reported as satisfactory [52].

The measurements of the metatarsus adductus angle according to the techniques presented by Root [3] and Simons [3, 46] showed significant differences by the first observer, 0.006–0.011, respectively, with ICC values of 0.945 and 0.935. The measurements of Berg [39] and Kilmartin [40] showed significant differences in intraobserver measurement by the second observer, 0.006 and 0.011, respectively, despite having high ICC values of 0.994 and 0.999.

Based on the results obtained and considering that there is no prior research comparing the different measurements, we believe that, in light of the results, the most reliable measurements for evaluating the metatarsus adductus angle are those developed by Laaveg and Ponseti [41], with high intraobserver ICC values of 0.982 and 0.999 and inter-observer ICC values of 0.995. Similarly, the rearfoot measurement [42–45] showed high intra-observer ICC values of 0.965 and 0.999, while presenting an inter-observer ICC of 0.991. These measurements also exhibited low values for SEM, CV, MDC, and no systematic differences were found between sessions on these two measures. We think it is very beneficial for clinicians to find out which measurement method to assess metatarsus adductus is more reliable to plan any kind of treatment and check the evolution of this pathology.

### Limitations of the study

For future research purposes, a weight-bearing computed tomography scan would be a valuable diagnostic tool for producing a 3D image of bones. Additional research is needed to determine the reliability and repeatability of metatarsus adductus angles [53]. Further research is needed to determine intra- and inter-observer radiographic metatarsus adductus in children assessed by experienced versus novel clinicians. Likewise, future studies need to determine the reliability and repeatability of metatarsus adductus angles as a measurement method used in other foot pathologies.

## Conclusion

We do not advise using Berg and Kilmartin’s measure for presenting intra-observer systematic differences nor do we recommend Root’s or Simons’ measures for presenting inter-observer systematic differences as well as their high MDC values.

All angle measurements used in the evaluation of radiographs of metatarsus adductus demonstrated high intra- and inter-observer reliability with ICC > 0.990, without showing systematic differences were the Laaveg & Ponseti and rearfoot measurements. Therefore, we recommend the use of these measurements.

## Data Availability

The datasets used to conduct this study will be available from the corresponding author upon reasonable request.
